# Touch me not! Jasmonic acid and ethylene converge on gibberellins breakdown to regulate touch-induced morphogenesis

**DOI:** 10.1093/plphys/kiad588

**Published:** 2023-10-31

**Authors:** Guadalupe L Fernández-Milmanda

**Affiliations:** Assistant Features Editor, Plant Physiology, American Society of Plant Biologists; Department of Plant Biotechnology and Bioinformatics, Ghent University, 9052 Ghent, Belgium; VIB, Center for Plant Systems Biology, 9052 Ghent, Belgium

Mechanical stimulation such as wind, rain, or touch induces a fast transcriptional reprogramming. When reiterated over time, this leads to a deep morphological restructuring, including growth repression and delay in flowering, a phenomenon known as thigmomorphogenesis (Braam and Davis 1990; Chehab et al. 2012; Braam and Chehab 2017). In the last years, 2 signaling branches emerged as regulators of touch-induced responses, which can be simplified as jasmonic acid (JA)–dependent or JA-independent signaling pathways (Chehab et al. 2012; Van Moerkercke et al. 2019; Darwish et al. 2022). In addition, catabolism of the growth-promoting hormone gibberellin (GA) is crucial to trigger thigmomorphogenesis (Lange and Lange 2015). However, it is not clear whether the JA (in)dependent singling branches regulate GA inactivation.

**Figure 1.**
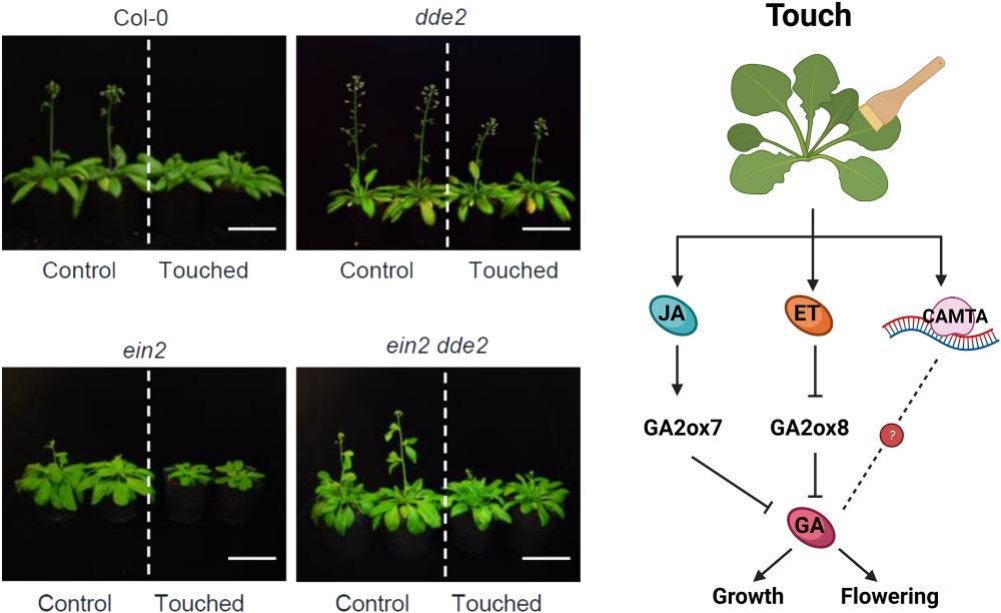
JA and ET converge on GA breakdown to regulate touch-induced morphogenesis, including growth repression and delay in flowering. Left and middle, photographs of Col-0 (wild type), *ein2* (ET-insensitive), *dde2* (JA biosynthesis mutant), and double *ein2 dde2* plants in control conditions or after repetitive touching treatment. Right, representative model of the molecular mechanisms controlling touch-induced morphogenesis. Touch treatment induces a complex signaling network, that can be divided into JA dependent and independent branches. JA activates the expression of *GA2ox7*, a gene coding for an enzyme involved in GA catabolism. In parallel, ET represses the expression of a close homologue of *GA2ox7*, *GA2ox8*, likely to prevent exaggerated responses. Besides hormonal regulation, CAMTA3 participates in transcriptional reprogramming in response to touch. An interesting lead for future research would be to see if CAMTA3 can also regulate GA breakdown, which would position GA metabolism as a central node in touch-induced morphogenesis. Adapted from Wang et al 2023. Created with BioRender.com.

In this issue of *Plant Physiology*, Wang et al. 2023 found 2 molecular pathways that regulate GA metabolism in response to touch, 1 dependent on JA and 1 dependent on an unexpected hormonal player, ethylene (ET). When the authors performed a transcriptomic analysis of plants gently touched with a paint brush, they noticed that genes related to ET biosynthesis and signaling were quickly induced. Although a previous study reported that touching treatment (in the form of gently rubbing the internodes of bean plants between thumb and forefinger) induces ET production (Biro and Jaffe 1984), ET signaling was not functionally linked to thigmomorphogenesis before. Thus, the authors characterized touch-induced morphogenesis in 2 ET-insensitive lines, *ein2* and *ein3 eil1*. As expected, repetitive touch repressed growth of wild-type plants, leading to more compact (reduced diameter) rosettes that also flowered later than their unstimulated counterparts. The *ein2* and *ein3 eil1* plants were also responsive to touch, but, interestingly, the effect of treatment was stronger than in the wild type, suggesting that ET could be a negative regulator of thigmomorphogenesis (Fig. 1, left).

Because breakdown of GA is necessary for expression of thigmomorphogenesis (Lange and Lange 2015), the authors measured levels of GA_4_, a bioactive GA, in the ET-insensitive lines. They found that touch treatment severely lowered the concentration of GA_4_ in the *ein2* and *ein3 eil1* plants, even to a greater extent than in the wild type. Furthermore, touch treatment induced the expression of *GA2ox8*, a gene coding for an enzyme involved in GA catabolism, exclusively in the *ein2* and *ein3 eil1* background. In agreement with these observations, electrophoretic mobility shift assays and in vivo transactivation assays using EIN3 and the promoter region of *GA2ox8* showed that EIN3 can directly bind the promoter of *GA2ox8* and inhibit *GA2ox8* transcription. Thus in the mutant lines, greater expression of *GA2ox8* likely leads to lower GA levels and a more compact rosette. Consistent with this model, supplementation of touch-induced *ein2* and *ein3 eil1* plants with exogenous GA_4_ rescued the compact growth and delay in flowering time phenotypes.

Touch treatment also induced the expression of GA2ox7, which is a homologue of GA2ox8 and has been previously linked to thigmomorphogenesis (Lange and Lange 2015). Because *GA2ox7* expression in the *ein2* and *ein3 eil1* plants was similar to that in the wild type, the authors concluded that this gene is not ET responsive. They then looked at the *GA2ox7* expression pattern in loss-of-function lines for JA biosynthesis (*dde2* line, which is a knock-out for the *ALLENE OXIDE SYNTHASE*; von Malek et al. 2002) or signaling (*myc234*, which is a multiple knockout for the MYC transcription factors; Fernández-Calvo et al. 2011). Both lines were previously shown to lack the thigmomorphogenic response (Chehab et al. 2012; Van Moerkercke et al. 2019). Consistent with prior results, touch treatment failed to induce *GA2ox7* expression in *dde2* and *myc234* plants, which coincided with a lack of GA_4_ breakdown. Transactivation assays with MYC2 and the promoter region of *GA2ox7* further suggested that MYC2 can activate *GA2ox7* expression, pointing out that JA controls thigmomorphogenesis by promoting GA2ox7-mediated GA catabolism.

Finally, to see if the ET and JA pathways interact, the authors generated an *ein2 dde2* double mutant. In response to repetitive touch, the *ein2 dde2* line showed neither the *ein2* hypersensitive phenotype (exacerbated growth repression and flowering delay) nor the *dde2* hyposensitive phenotype (no growth repression or flowering delay). Rather, the *ein2 dde2* plants responded like the wild type (Fig. 1, middle). Consistently, GA4 levels in the *ein2 dde2* plants were comparable to those in the wild-type plants. Finally, *GA2ox7* expression was equivalent between the *dde2* and the *ein2 dde2 lines* and *GA2ox8* expression was equivalent between the *ein2* and the *ein2 dde2* plants, suggesting that ET and JA regulate thigmomorphogenesis by 2 independent but additive molecular mechanisms that converge in the regulation of GA catabolism (Fig. 1, right).

In conclusion, Wang and collaborators explored the connection between the pathways of 2 known regulators of touch-induced morphogenesis, JA and GA. They found that JA activates GA breakdown, leading to decreased growth and delayed flowering. The authors also incorporated ET into the picture. ET acts as a negative regulator of thigmomorphogenesis, also through an effect on GA catabolism, but in this case ET represses GA catabolism, likely to prevent exaggerated responses (Figure 1). These findings position GA metabolism as a central node in thigmomorphogenesis.

Previous studies have shown that CALMODULIN-BINDING TRANSCRIPTION ACTIVATOR 3 (CAMTA3) also participates in transcriptional reprogramming in response to touch (Darwish et al. 2022; Matsumura et al. 2022). This pathway represents another JA-independent signaling branch. Thus, future research may address if CAMTA3 also is involved in regulating the expression of genes coding for GA2OX.
